# Improvement of Cryopreservation and Production of Attenuated *Babesia* Parasites to Prevent Bovine Babesiosis

**DOI:** 10.3390/pathogens14050498

**Published:** 2025-05-20

**Authors:** Carmen Rojas-Martínez, José J. Lira-Amaya, Massaro W. Ueti, Roberto O. Castañeda-Arriola, Julio V. Figueroa Millán, Jesús A. Álvarez Martínez

**Affiliations:** 1 *Babesia* Unit-CENID-Salud Animal e Inocuidad, INIFAP, Carretera Federal, Cuernavaca-Cuautla No. 8534, Col. Progreso, Jiutepec C.P. 62550, Morelos, Mexico; rojas.carmen@inifap.gob.mx (C.R.-M.); lira.juan@inifap.gob.mx (J.J.L.-A.); castaneda.roberto@inifap.gob.mx (R.O.C.-A.); figueroa.julio@inifap.gob.mx (J.V.F.M.); 2 Agricultural Research Service-Animal Disease Research Unit, The US Department of Agriculture, Pullman, WA 99164, USA

**Keywords:** cryopreservation, *Babesia bigemina*, *B. bovis*, babesiosis vaccine

## Abstract

This study evaluated the effects of various concentrations of PVP-40 on the in vitro cryopreservation and recovery of *Babesia bovis* and *Babesia bigemina*. We also assessed a reduced dose of attenuated *Babesia* strains to determine its efficacy in preventing clinical disease. A microaerophilic stationary phase blood culture system was used to recover *Babesia* parasites that were cryopreserved in solutions with various PVP-40 concentrations and *Babesia* parasites in 20% PVP-40 were used to vaccinate naïve cattle. The animals were vaccinated intramuscularly with frozen parasites cryopreserved in 20% PVP-40, with a dose of either 1 × 10^8^ or 1 × 10^7^ erythrocytes infected with both attenuated *B. bigemina* and *B. bovis* produced from blood cultures. The control group received uninfected erythrocytes. During the vaccination, clinical parameters such as rectal temperature and hematocrit levels were unaffected. The animals were relocated to a farm in a *Babesia* hyperendemic area to test the efficacy of these live vaccines in controlling disease onset. Some vaccinated animals showed mild disease. In the vaccinated groups, parasites were detected in blood smears for only one day during the challenge. In contrast, the control group experienced fever for three consecutive days, a decline in hematocrit levels, and significant health deterioration. In this group, parasites were detected in smears for four consecutive days. All the animals in the control group required treatment to manage their high parasitemia and prevent mortality. In this study, we demonstrated that increasing the concentration of PVP-40 to cryopreserve parasites improved the recovery and proliferation of *Babesia* spp. in blood culture, and we also showed that when animals were vaccinated with cryopreserved, in vitro cultured, attenuated *Babesia* parasites in 20% PVP-40, they were effectively protected from severe clinical babesiosis.

## 1. Introduction

*Babesia bovis* and *Babesia bigemina* are intraerythrocytic apicomplexan parasites that are responsible for bovine babesiosis, which is transmitted by the ticks *Rhipicephalus microplus* and *Rhipicephalus annulatus*. The clinical signs of babesiosis include fever, hemolytic anemia, hemoglobinuria, anorexia, and, in many cases, death [1,2]. This disease significantly impacts livestock in tropical and subtropical regions of the world, leading to decreased milk and meat production [3,4].

The cultivation of *B. bovis* or *B. bigemina* using a microaerophilic stationary phase culture system (MASP) is a crucial methodology with wide-ranging applications [5]. Once established in an in vitro blood culture, these parasites continuously proliferate, providing valuable insights into their biology, nutritional needs, and therapeutic potential [6]. This approach enhances drug evaluation [7], facilitates parasite cloning, and supports the development of live attenuated vaccines [8,9]. After the culture of *B. bovis* and *B. bigemina* have been established, the parasites are mixed with a cryoprotectant and then stored in liquid nitrogen. This preservation method enables the subsequent production of antigens and vaccines [10]. Moreover, the MASP technique has remarkably enhanced our ability to sustain multiple new field isolates of *Babesia* parasites in the laboratory, all while keeping costs low [11].

Several key factors influence the viability of cells or microorganisms during cryopreservation, including the type and concentration of the cryoprotectant as well as the freezing and thawing processes [12]. Previous studies have indicated that polyvinylpyrrolidone-40 (PVP-40), a nontoxic, neutral hyprophilic polymer belonging to extracellular cryophylactic agent group, is the least toxic extracellular cryoprotectant for *B. bovis* [13,14]; similar findings have been observed for *B. bigemina* [15]. This protocol has long been used as a standard; however, it has led to poor recovery of infected erythrocytes and low viability of the parasites. As a result, it has been challenging to collect sufficient biological material, which complicates the recovery of previously cryopreserved *Babesia* parasite isolates.

We have reported the use of in vitro cultures to produce material for live vaccines against babesiosis, which have demonstrated a high level of protection in susceptible cattle during natural challenges. The standard dose protocol is 1 × 10^8^ infected erythrocytes from each species [16]. Nevertheless, it would be beneficial to use a smaller quantity of infected erythrocytes as a vaccine dose to prevent severe bovine babesiosis. Using a log lower number of infected erythrocytes in the vaccine offers a significant advantage in terms of cost and time. In practice, preparing a batch of 10 doses with 1 × 10^8^ infected erythrocytes convert to 100 doses of 1 × 10^7^ infected erythrocytes, which provides a more noticeable benefit on larger scales. The aim of this study was to assess the effects of various concentrations of PVP-40 on the cryopreservation and recovery of *B. bovis* and *B. bigemina* under in vitro conditions, as well as to prepare a reduced-dose vaccine of 1 × 10^7^ infected erythrocytes and evaluate its effectiveness under field conditions.

## 2. Materials and Methods

### 2.1. Site of Field Challenge

This study focused on the vaccination and subsequent challenge of cattle under field conditions. During the vaccination period, the animals were kept on a tick-free farm for three weeks. Following this, a natural challenge was conducted by moving the cattle to a farm where the *Rhipicephalus microplus* ticks, as well as *B. bovis* and *B. bigemina* parasites, are endemic. The farms are located in the state of Veracruz, Mexico.

### 2.2. Animals

To obtain erythrocytes for in vitro *B. bovis* and *B. bigemina* culture, a five-year-old uninfected Holstein Friesian bovine was used as a blood donor. Blood was collected from the jugular vein, defibrinated with glass beads, and centrifuged at 450× *g* at 4 °C for 30 min. The erythrocytes were separated, and the serum and buffy coat were discarded. The collected erythrocytes were washed three times with A-DMEM/F12 + a mixture of antioxidants (*v*/*v*) and suspended in the same solution in a 1:2 proportion (*v*/*v*), and then stored at 4 °C until use [17].

For vaccination and challenge, 17 one-and-a-half-year-old Holstein Friesian cattle were used. The cattle were raised in an area free of ticks and tested negative for bovine brucellosis, tuberculosis, leucosis, IBR, BDV, *Anaplasma marginale*, and *Babesia* spp. Furthermore, they were handled according to the NOM-062-ZOO-1999 technical specifications for production care and use of laboratory animals, accessed on 1 May 2022 (https://www.gob.mx/senasica/documentos/nom-062-zoo-1999).

### 2.3. Culture Media and Supplements

The culture medium used was VP-SFM (Gibco, Grand Island, NE, USA) with CD-Lipid (Gibco^®^) buffered with 25 mM 2-[(2-hydroxy-1, 1-bis (hydroxymethyl)ethyl) amino] ethane sulfonic, N-[Tris (hydroxymethyl)methyl]-2-aminoethanosulphonic (TES) (Sigma-Aldrich, St. Louis, MO, USA) and 2 mM L-glutamine (Gibco^®^) and an antioxidant mixture (Sigma-Aldrich, St. Louis, MO, USA) were added; the pH was adjusted to 6.8. The cryoprotectant polyvinylpyrrolidone-40 (PVP-40) (Sigma-Aldrich, St. Louis, MO, USA) was used at percentages of 20%, 30%, 40%, and 50% (*w/v*) in A-DMEM/F12 medium. All the solutions used were sterilized by filtration with 0.22 µm membranes [18].

### 2.4. In Vitro Blood Culture of Parasites

The Bbig-SF *B. bigemina* strain and Bbov-SF *B. bovis* strain were utilized in this study. These Mexican *Babesia* strains were clones derived from an in vitro culture that were adapted to a culture medium without bovine serum for more than 5 years [6]. The continuous culture of the individual *Babesia* species started in 24-well tissue culture plates, which was then expanded to 75 cm^2^ culture flasks with A-DMEM/F12. The cultures were maintained at 37 °C in a saturated atmosphere with a gas mixture of 90% N_2_, 5% CO_2_, and 5% O_2_ [18]. The culture medium was replaced every 24 h with fresh medium and a 10% suspension of erythrocytes in A-DMEM/F12 (*v*/*v*). The percentage of parasitized erythrocytes was estimated every 24 h from Giemsa-stained blood smears and 5000 total erythrocytes were examined to determine the parasitemia. Three replicates of the experiments and three repetitions of each treatment were carried out.

### 2.5. Cryopreservation

The *B. bigemina* and *B. bovis* strains were proliferated individually in a 75 cm^2^ culture flask; the erythrocyte packets were collected and centrifuged at 450× *g* at 37 °C for 30 min. The supernatant was discarded and PVP-40 was added to obtain final concentrations of 10%, 15%, 20% and 25% in DMEM/F12 medium. Two milliliters of packed erythrocytes with a parasitemia of 5% were mixed at a ratio of 1:2 (*v*/*v*) with each of the PVP-40 concentrations. Immediately after the cryoprotectant was added to the erythrocytes, the mixture was gently homogenized to integrate the PVP-40 into the erythrocytes. They were subsequently frozen at −70 °C where they remained for 24 h before being transferred to liquid nitrogen (−196 °C). Each treatment was performed with three repetitions and three replicates. Subsequently, the proliferation of the parasites after thawing was determined in the mixtures with different concentrations of the cryoprotectant.

### 2.6. Recovering Frozen Babesia Bigemina and Babesia bovis from In Vitro Blood Culture

The *B. bigemina* or *B. bovis* cryopreserved in 10%, 15%, 20% and 25% PVP-40 were kept in liquid nitrogen for six months. The samples were thawed rapidly at 37 °C with gentle shaking and then transferred to 50 mL tubes containing 45 mL of 37 °C A-DMEM/F12 and centrifuged at 450× *g* at 37 °C for 30 min. The pellet of each sample was suspended in 1 mL of A-DMEM/F12 and transferred to 24-well plates to start the in vitro culture. The culture medium was replaced every 24 h and the first subculture was performed at 96 h; the parasitemia was adjusted to 1% with a 10% erythrocyte suspension in A-DMEM/F12 medium (*v*/*v*). The bovine babesiosis attenuated vaccines were made with the Bbig-SF *B. bigemina* or Bbov-SF *B. bovis* strains that were cultivated separately and thawed just before they were injected into the experimental bovines, as previously described [6].

### 2.7. Antigen Production

Separate suspensions of *B. bovis*- and *B. bigemina*-infected erythrocytes were obtained from the in vitro cultures. When the percentage of parasitized erythrocytes (PPE) was at least 5%, each suspension was transferred to 50 mL tubes and centrifuged at 450× *g* for 30 min at 4 °C. The supernatant was removed, and the pellets were resuspended in 45 mL of A-DMEM/F12, followed by three centrifugation washes. From the resuspended erythrocyte packet, 7 µL was taken and spread onto a glass slide, air-dried, and wrapped with absorbent paper and aluminum foil. These samples were then stored at −20 °C until they were used for the immunofluorescence assays.

### 2.8. Indirect Fluorescent Antibody Test

Serum samples were collected from the experimental groups and the local herd where the challenge was performed. The indirect fluorescent antibody test (IFAT) was performed as previously described [19]. Slides were coated with 5% B. bovis- or B. bigemina-infected erythrocytes from separate in vitro cultures. They were dried and fixed with an acetone solution for 15 min. Each bovine serum sample used for diagnosis and titration was diluted 1:80 in PBS (pH 7.2), placed on slides, and then incubated at 37 °C for 30 min and washed three times with PBS. After that, Alexa Fluor 488-conjugated goat anti-bovine IgG (Jackson Immuno Research Laboratories, Inc., West Grove, PA, USA) was diluted 1:2000 in PBS and added to each sample, which was then incubated at 37 °C for 30 min and washed three times with PBS. The microscopic detection of positive reactions was carried out using an epifluorescence LEICA DMLB microscope. Positive and negative reference sera were included on each slide.

### 2.9. Parasite Detection PCR

Parasite detection in the peripheral blood of animals and in the ticks was performed using nested PCR (nPCR). In addition to the 17 experimental animals, 130 blood samples from the local herd were collected to determine the infection rates. Briefly, blood from the bovine was collected via caudal venipuncture using Vacutainer tubes with EDTA (BC Company, Flanklin Lakes, NJ, USA). Genomic DNA extraction was performed from packed red blood cells using the commercial Quick-DNA Miniprep Kit (Zymo Research, Orange, CA, USA) following the manufacturer’s instructions. The specific primers listed in Table 1 were used to amplify 275 and 412 bp fragments of the *Babesia bovis* rap-1 and *B. bigemina* rap-1a genes, respectively [20,21]. Both single and nPCR protocols were carried out in a final volume of 25 µL; the mixture included 12.5 µL of Go 2x Taq Green Master Mix (400 µM of dNTP, 3 µM MgCl2, and 1.5 U Taq DNA polymerase in 2× buffer, pH 8.5; PROMEGA, Madison, WI, USA), 1 µL of each of the forward and reverse primers (10 pmol), and 80–120 ng of genomic DNA as the template for a single PCR reaction and 2 µL of the generated amplicon as the template for nPCR. PCR-grade nuclease-free water was used to adjust the final volume to 25 µL. The reaction conditions for the rap1-B. *bovis* PCR were 95 °C for 5 min, 35 cycles of 95 °C for 1 min, 55 °C for 1 min, 73 °C for 1.5 min, and extension at 72 °C. The reaction conditions for the rap-1a-*B. bigemina* PCR were 95 °C for 5 min, 35 cycles of 94 °C for 1 min, 55 °C for 1 min, and extension at 72 °C. The amplicons were analyzed by 2% agarose gel electrophoresis, stained with ethidium bromide, and then visualized using ultraviolet light.

To detect *Babesia* in ticks, engorged adult female ticks were collected from 40 animals on the farm where the challenge was conducted. For DNA extraction from R. microplus, each tick was individually macerated in a microtube in 600 µL of PBS and centrifuged at 800× *g* for 15 min. Twenty microliters of supernatant was used for the DNA extraction. The PCR conditions described above were used to detect *B. bovis* or *B. bigemina* in the ticks.

### 2.10. Vaccination and Challenge

Seventeen cattle were randomly assigned into three groups to test if the cryopreserved, attenuated *Babesia* parasites in 20% PVP-40 can protect animals exposed to the tick vector in an endemic field. Group I consisted of six animals injected with a vaccine containing 1 × 10^8^ erythrocytes infected with both *Babesia* species (*B. bigemina* and *B. bovis*). Group II included six animals vaccinated with a lower dose containing 1 × 10^7^ erythrocytes infected with both species. The vaccines containing 20% PVP-40, culture medium, and infected red blood cells were thawed and intramuscularly injected into the animals. The same doses of *B. bovis* and *B. bigemina* were used, which were inoculated at the same time. Group III comprised five animals intramuscularly inoculated with 1 × 10^8^ uninfected bovine erythrocytes and served as the control group. All cattle were kept on a ranch that was free of ticks and *Babesia*, located at the coordinates 19°38′14″ N and 97°05′54″ W. After this period, the animals were moved to tick-infested farms located at the coordinates 19°2′6′′ N and 96°8.133′ W, where they were exposed to R. microplus ticks infected with *B. bovis* and *B. bigemina*. No acaricide treatment was administered during the challenge experiment.

During vaccination and challenge, all the animals were monitored daily for severe clinical signs of bovine babesiosis, including lethargy, weakness, depression, fever (rectal temperature), loss of appetite, anemia, and weight loss. In addition, blood samples were collected daily to monitor the packed cell volume based on the hematocrit (%) and parasite detection was performed using microscopic examination of Giemsa-stained blood smears. An animal was considered to have severe acute disease if it simultaneously showed the following symptoms for three consecutive days: (1) a 25% reduction in hematocrit compared to baseline; (2) a rectal temperature above 40 °C; and (3) *Babesia* spp. were detected in the Giemsa-stained blood smears. These parameters were used to determine if babesicidal treatment should be administered by intramuscular injection (3.5 mg/kg of 4,4′-diazoaminodibenzamidine diacetate for three consecutive days) to prevent death and the animal was recorded as an experimental death.

### 2.11. Statistical Analysis

The data were analyzed using the median percentage of parasitized erythrocytes for each group by applying the nonparametric Kruskal–Wallis test. Additionally, we assessed the independence of vaccination and clinical disease protection in the experimental animals using Fisher’s exact test (*p* < 0.05). Furthermore, we generated descriptive statistics and analyzed variables with two or three categories for antibody titration, rectal temperature, and hematocrit levels.

## 3. Results

### 3.1. Effect of PVP-40 Concentration on the Recovery of Parasites

**Figure 1:**
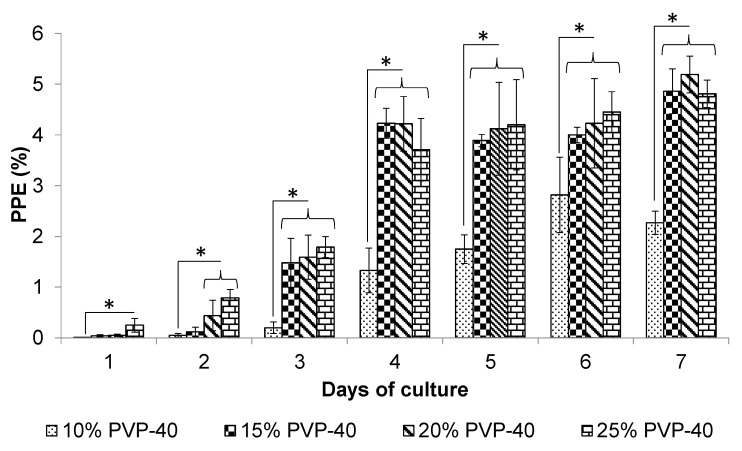
The effect of PVP-40 concentration on the in vitro recovery of *B. bigemina*. The percentage of parasitized erythrocytes (PPE) was assessed through blood smear analysis. The error bars indicate the standard deviation. Significant differences were observed between the PVP-40 treatment groups and 10% PVP-40 control group (* = *p* < 0.05).

Increasing the concentration of PVP-40 for cryopreservation resulted in faster recovery and higher parasitemia for both *B. bigemina* and *B. bovis* in vitro blood culture compared to using 10% PVP-40. On day 3, the *B. bigemina* cultures exhibited significantly higher growth (*p* < 0.05) compared to the 10% PVP-40 control (Figure 1). By day 4, the recovered parasites reached over 3.8% parasitemia. In contrast, the cryopreserved parasites in the 10% PVP-40 group grew more slowly. By day 7, the cryopreserved *B. bigemina* in 20% PVP-40 achieved the highest parasitemia, with a parasitemia exceeding 5.1%, while the 10% PVP-40 control showed only a parasitemia of 2.3% (Figure 1).

**Figure 2:**
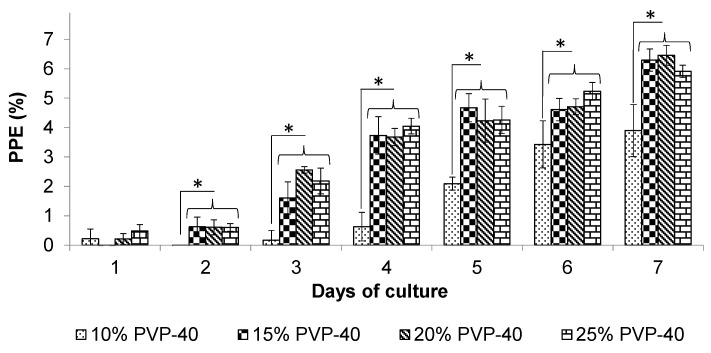
The effect of PVP-40 concentration on the recovery of *B. bovis* in in vitro cultures. The recovery of *B. bovis* was examined by measuring the percentage of parasitized erythrocytes (PPE) through blood smear analysis. The error bars indicate the standard deviation. Significant differences were observed between the PVP-40 treatment groups and 10% PVP control group (* = *p* < 0.05).

The recovery and proliferation of *B. bovis* that was cryopreserved with higher concentrations of PVP-40 mirrored those of *B. bigemina*. On day 3, the *B. bovis* cultures also demonstrated significantly higher growth (*p* < 0.05) compared to the 10% PVP-40 control, indicating a rapid recovery of *B. bovis* in the in vitro blood cultures. By day 4, the recovered parasites again surpassed 3.8% parasitemia, while the cryopreserved parasites in the 10% PVP-40 group only reached 0.6% parasitemia (Figure 2). On day 7, the cryopreserved *B. bovis* in 20% PVP-40 showed the highest parasitemia at 6.6%, while those recovered from the 10% PVP-40 control remained below 4% (Figure 2). We observed similar recovery and proliferation rates for *B. bigemina* and *B. bovis* in the in vitro blood cultures.

### 3.2. Prevalence of Babesia Parasites and Tick Infection Rate

The local herd where the challenge was performed consisted of 130 cattle and the seroprevalence was 90% for *B. bovis* and 95% for *B. bigemina* according to the IFAT. The prevalence of Babesia infection according to nPCR was 35% and 57% for *B. bovis* and *B. bigemina*, respectively. Moreover, 290 engorged R. microplus female ticks were collected from the same animals. The infection rate based on nPCR was 12% and 14% for *B. bovis* and *B. bigemina*, respectively.

### 3.3. Vaccinated Animals

Rectal temperature. No cases of fever were detected on any day. The overall average body temperature of all the animals in the three groups was 37.9 °C.

Detection of *B. bovis* and *B. bigemina* by nPCR. *B. bovis* and *B. bigemina* were detected in peripheral blood samples a few days after vaccination using nPCR. On day 8 after vaccination, in Group I, *B. bovis* was detected in four animals and *B. bigemina* was detected in three animals. In Group II, *B. bovis* was detected in three animals and *B. bigemina* was only detected in one animal. Subsequently, on day 17 after vaccination, the number of animals with vaccine-derived parasites increased. In Group I, five animals were positive for *B. bovis* and five animals were positive for *B. bigemina*. In Group II, three positive animals for each *Babesia* species were detected. *Babesia* parasites were not detected in any of the Group III animals during the vaccination phase. On day 17, coinfection with *B. bovis* and *B. bigemina* was detected in four and three animals in Group I and Group II, respectively (Table 2).

Detection of parasitized erythrocytes. In Group I, *Babesia* parasites were detected in two animals on day 8 using microscopic examination of Giemsa-stained blood smears. By day 12, parasites were detected in all animals in this group, with a parasitemia of approximately 0.002%. In Group II, circulating parasites were identified in two out of six cattle on day 12 and they had a parasitemia of 0.001%.

Packed cell volume. The normal packed cell volume values remained unaffected by the application of the vaccine in both Group I and Group II, with an average hematocrit of 33.8%.

IgG responses to *B. bovis* and *B. bigemina*. Seroconversion was observed on day 8 in the vaccinated Group I and Group II for both *B. bovis* and *B. bigemina*. The unvaccinated cattle in Group III remained negative during this phase. At the end of the vaccination phase, the titers of antibodies in Group I and Group II vaccinated animals were above 1:2000 for *B. bovis* and *B. bigemina*.

### 3.4. Challenged Animals

**Figure 3:**
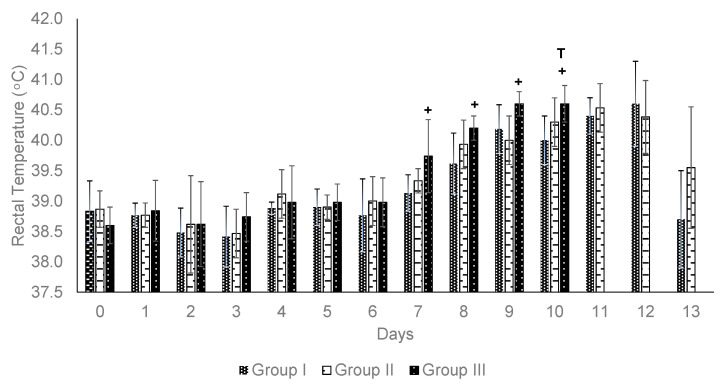
Rectal temperature recording during field challenge of vaccinated cattle. + indicates positive blood smear for *Babesia* parasites. T: drug treatment animals suffering severe clinical bovine babesiosis.

Rectal temperature. Fever was observed in some animals during the challenge phase. In Group I, one out of six animals developed a fever on day 7, and four out of six cattle exhibited fever on day 10, with an average temperature of 39.9 °C. In Group II, three out of six animals experienced fever on day 8, and this increased to four out of six on day 10, with an average temperature of 39.7 °C. Notably, in both vaccinated groups, no animal experienced fever for three consecutive days, and their temperature returned to normal values by day 13. In Group III, which consisted of unvaccinated animals, three out of five animals had a fever on day 7. By day 8, all animals in Group III exhibited fever, with an average temperature of 40.2 °C. Consequently, from day 7 to 10, the average body temperature remained above 40 °C (Figure 3).

Detection of *B. bovis* and *B. bigemina* by nPCR. On day 8 of the field challenge, *B. bovis* was detected in all animals in all three groups. On the same day, *B. bigemina* was detected in two animals in Group I and Group II. In contrast, *B. bigemina* was not detected in any animal in Group III during this period. Subsequently, on day 13, we detected *B. bovis* infection in five animals in Group I, six animals in Group II, and five animals in Group III. We also found that on day 13 of the challenge, four animals in Group I, three animals in Group II, and four animals in Group III were positive for *B. bigemina*. Coinfection with *B. bovis* and *B. bigemina* was detected in three animals in Group I, two animals in Group II, and three animals in Group III (Table 3).

**Figure 4:**
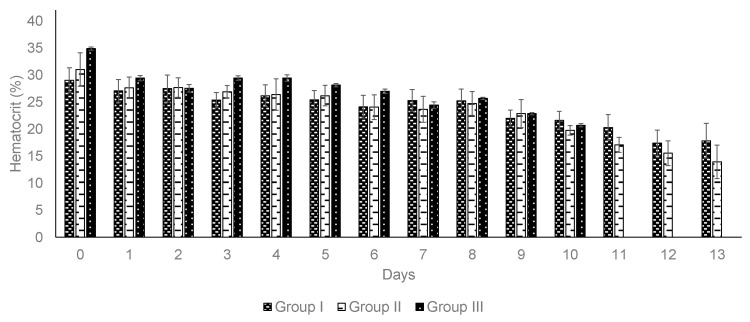
Hematocrit recording during field challenge phase. All animals were not treated with ixodicides during the field challenge. The error bars indicate the standard deviation. All animals were challenged with natural exposure under field conditions.

Packed cell volume. Group I and Group II experienced reductions in hematocrit of 24.1% and 35.5%, respectively (Table 4). Despite the ongoing decline in hematocrit levels (Figure 4), all the animals in the vaccinated groups did not exhibit any changes in their health conditions. In contrast, Group III showed a hematocrit reduction of 40.0% (Table 4). All the animals in Group III experienced severe clinical signs of babesiosis, including fever, increased respiratory rate, weakness, lethargy, ruminal atony, weight loss, impaired ambulation, and prostration.

IgG responses to *B. bovis* and *B. bigemina*. Animals in Groups I and II remained positive for *B. bovis* and *B. bigemina*. From the sampling carried out when introducing the animals to a farm with high endemicity for *Babesia* spp., Group I had a higher antibody titer for *B. bovis*, with an average of 1:4693, but for *B. bigemina*, the average titer was 1:2240. In Group II, the average titer for B. bovis was 1:2287 and for *B. bigemina*, it was 1:2400. In Group III, no antibody response was observed because the animals had not been previously exposed to *Babesia* parasites.

On day 7 of the challenge, the animals in Group I showed a stronger humoral response, with an average antibody titer of 1:10,240 for *B. bovis* and 1:5973 for *B. bigemina*. In Group II, the average antibody titers were similar to those of the first sampling, with values of 1:2560 for *B. bovis* and 1:2304 for *B. bigemina*. Group III showed a moderate immune response seven days after their first exposure to *Babesia* spp. in the natural challenge. The average antibody titer achieved for *B. bovis* was 1:208 and for *B. bigemina*, it was 1:352.

Protection against bovine babesiosis. In Group I, parasites were observed in three animals based on the Giemsa-stained blood smears. In Group II, parasites were observed in four animals (Table 4). For both groups, detection occurred on day 10 of the challenge. In Group III, parasites were detected in all animals on day 7 (Table 4). The presence of parasites, along with the symptoms of severe bovine babesiosis and four consecutive days of fever, underscored the urgent need for drug treatment to prevent high parasitemia and mortality. In Group III, the animals were treated with diminazene aceturate at a dosage of 3 mg/kg and subsequently removed from the experiments. Protection was not independent of vaccination. There was a significant difference between Group I and Group II versus Group III (*p* < 0.05) (Table 4).

## 4. Discussion

In this study, we demonstrated that parasitemia after recovery from cryopreservation was improved by using a higher concentration of PVP-40 for both *B. bovis* and *B. bigemina*. Additionally, we found that administering a vaccine dose of 1 × 10^7^ *Babesia*-infected erythrocytes cryopreserved in 20% PVP-40 provided protective effects for cattle exposed to a natural challenge under field conditions.

Cryopreservation and recovery of *Babesia* parasites. Polyvinylpyrrolidone-40 (PVP-40), a nontoxic, neutral, hyprophilic polymer, is the least toxic extracellular cryoprotectant for *Babesia* parasites [14]. In this study, the best recovery values for cryopreserved *Babesia* parasites were obtained using PVP-40 in a final concentration of 15% to 25%. Previously, PVP-40 with VYM was used at a final concentration of 10% for cryopreservation of *Babesia* spp. instead of the 15–25% PVP-40 in DMEM/F12 medium and bovine serum used in this study. The addition of high-concentration serum has been reported to improve cryopreservation recovery [22]. Our data demonstrated that higher final concentrations of PVP-40 between 15% and 25% improved the recovery and proliferation of frozen parasites in vitro blood culture. Cryoprotective agents are typically added for cryopreservation, but interestingly, only a few solutions have been evaluated for erythrocytes [23], and even fewer for *Babesia*-infected erythrocytes. For human red blood cells, a mixture of glucose, PVP-40, and human serum albumin has been recommended to protect membrane integrity after cryopreservation [24]. PVP-40 has been shown to be an effective cryoprotectant for *B. bovis*, *B. bigemina*, and *Anaplasma centrale*; it is similar to DMSO but is less toxic to the parasites [25]. Some combinations of cryoprotectants, such as 10% cell membrane-penetrating DMSO and 20% non-penetrating polyethylene oxide (PEO-1500), have been shown to be more effective in protecting bovine red blood cells at different stages of cryopreservation compared to the use of a single product such as DMSO [26].

For the cryopreservation of *B. gibsoni*, which cause babesiosis in dogs, 20% PVP-40 has been reported as the highest effective concentration; however, after diluting the infected red blood cell pack 1:2 (*v*/*v*), the final PVP-40 concentration was only 10% [27]. Likewise, for *B. bigemina*, a final concentration of 10% PVP-40 was used as a cryoprotectant [15]. Therefore, a major difference in this study was the final concentration of PVP-40, which was found to improve the in vitro recovery. Standardization of the cryopreservation method might not only be necessary to ensure recovery but also a sufficient transfection efficiency for research activities [28]. The rapid recovery of *B. bovis* and *B. bigemina* parasites makes their establishment in in vitro cultures and in animals during vaccination more efficient and effective.

Vaccination and Challenge. To test if vaccinating with frozen, attenuated *Babesia* parasites in 20% PVP-40 can provide protection against severe bovine babesiosis, we vaccinated cattle and challenged them by placing them in a highly endemic area for bovine babesiosis. During the vaccination phase, no changes were observed in the rectal temperature or packed cell volume for both doses (1 × 10^8^ and 1 × 10^7^) of *B. bovis*- and *B. bigemina*-infected erythrocytes when inoculated intramuscularly. Circulating parasites were detected on day 7 using Giemsa-stained blood smears and confirmed by nPCR. Other studies found that when using a vaccine at a lower dose of 1 × 10^6^ that was administered intravenously, the animals showed mild signs of disease and the recovery of temperature and packed cell volume to normal values was observed without anti-Babesia treatment [29,30].

In our study, during the field challenge, the vaccinated animals showed mild clinical signs, but treatment was not necessary because neither the fever nor the presence of circulating parasites persisted for three consecutive days, and the animals did not suffer severe acute babesiosis. Unlike the control group, the animals showed the typical signs of acute babesiosis, including lethargy, weakness, depression, fever for more than three consecutive days, loss of appetite, anemia, and weight loss. Drug treatments were administered to all the animals in the control group to prevent death. Similar observations during a challenge were also reported by Bastos et al. [29,30]. However, there were important differences. In Bastos et al. [29,30], the vaccination with *B. bovis* (Att-S74-T3Bo) and challenge with *B. bovis* (Vir-S74-T3Bo) were administered intravenously, so there was no natural exposure to *Babesia*-infected ticks and *B. bigemina* was not included in those studies. In our study, the animals were exposed to *Babesia* spp. in a field under natural conditions rather than laboratory-controlled conditions. Regarding live attenuated vaccines, an interesting and promising scientific innovation has been reported. The use of gene-edited, in vitro cultured *B. bovis* has been described, which was used as a vaccine in cattle and allowed for the expression of exogenous proteins such as GST or Bm86 tick antigens. In this way, dual vaccine materials could be administered for the control of *Babesia* infection and tick infestation [31,32]. Although a challenge was not indicated, mild signs of babesiosis were described, including fever and a decreased packed cell volume, after vaccination [32]. All the animals fully recovered without drug intervention. In contrast, in our study, after vaccination, no alterations were observed in the rectal temperature or in the hematocrit, and the parasitemia was at least 50 times lower than the transfected parasites expressing BM86. There are several explanations for these differences, including the different routes of immunization (intramuscular versus intravenous) and different *B. bovis* strains used in the experiments.

In our study, the hematocrit continuously declined during the field challenge; this could be attributed to the fact that the animals were not treated with acaricides to control tick infestation. The combined effect of tick infestation and *Babesia* infections was found to be detrimental, but moderate, in the vaccinated groups. In the control group, severe clinical bovine babesiosis disease was observed during the field challenge and all the animals in this group received drug treatment. Currently, in vitro cultured vaccines against *B. bovis* and *B. bigemina* are produced under standardized and controlled conditions, with reduced risks of contamination by adventitious agents [33]. Live attenuated vaccines are currently essential and the only available option for preventing clinical outbreaks. However, maintaining the long-term immunogenicity of in vitro cultured *Babesia* spp. parasites will be crucial [34]. Moreover, the use of attenuated vaccines has the advantage of inducing enzootic stability.

While vaccines based on recombinant proteins have demonstrated the ability to induce an immune response to pathogens like *B. bovis* and *B. bigemina*, they often provide limited or no protection during clinical outbreaks. Therefore, continued research is essential for designing an effective, safe, and efficient vaccine for controlling bovine babesiosis. To practically and objectively evaluate an attenuated vaccine, its benefits in terms of protection should be assessed through clinical findings and cattle mortality rates. It is important to note that developing a subunit vaccine against bovine babesiosis poses significant challenges for vaccinology. Despite noteworthy advancements in immunology, bioinformatics, and omics, the results achieved so far have been promising yet inconclusive [35].

The humoral response at the end of the vaccination phase showed that the vaccinated animals in Group I and Group II rapidly developed significant levels of IgG against *B. bovis*. A similar response was described in a vaccination study using an attenuated strain of *B. bovis* that was cultured in vitro and inoculated into adult animals under controlled conditions [30]. However, unlike our study, an indirect ELISA using the C-terminal segment of *B. bovis* rhoptry-associated protein 1 (RAP-1CT) as the antigen showed that the maximum antibody response occurred 15 days post-vaccination [29]. Another study with Bos indicus calves maintained in a farm with enzootic stability for babesiosis showed antibody titers similar to those in our study (1:3000 to 1:5000 for both *B. bovis* and *B. bigemina*). However, this finding was observed after a decrease in passive immunity after ingesting colostrum, so an increase was noted between three and eight months of age [36].

The IgG antibody levels from the start of inoculation to day 7 showed a considerable increase, especially in Group I. This can be interpreted as a classic secondary immune response given the short period and the high titer achieved. However, in Group II, a secondary immune response was not detected for either *Babesia* species. The antibody titers were essentially similar to those measured at the start of challenge. In contrast, another study showed that animals vaccinated with an attenuated strain of *B. bovis* and challenged with a virulent strain did not exhibit the booster effect seen in our study. Although unvaccinated animals succumbed to the acute form of babesiosis, this occurred between days 10 and 12 of the challenge [30]. This differs from the seven days observed for the unvaccinated group in this study. This may be explained by the fact that the challenge was a natural tick infestation on a ranch where the local herd had a high prevalence of *B. bovis* and *B. bigemina* and ixodicides were not used. In addition, we used naive Holstein Friesian animals, which are highly susceptible to tick infestation and to the clinical form of babesiosis.

The nPCR assay was useful for verifying the presence of one or both *Babesia* species in vaccinated animals. During the challenge, nPCR identified circulating parasites not only in vaccinated animals from Group I and Group II, but also in unvaccinated animals from Group III. We observed that the *B. bovis* infection rate was higher than the *B. bigemina* infection rate on day 8, and on day 13, all the animals tested positive for one or both *Babesia* species. Unfortunately, nPCR was not able to differentiate the vaccine strains from the wildtype strains in the Group I and Group II animals. Nonetheless, the nPCR assay was able to inform us if the animals in Group III were infected with *B. bovis*, *B. bigemina*, or both parasites. It is important to note that currently available vaccines against bovine babesiosis are primarily based on live attenuated parasites derived from multiple passages in splenectomized calves or in vitro cultures. These vaccines have demonstrated very favorable protection against virulent strains after natural exposure [3].

In general, the development of vaccines against *B. bovis* and *B. bigemina* has mainly focused on live attenuated parasites, recombinant proteins (either individually or in cocktails), and antigenic peptides. For *B. bovis* in particular, the molecular mechanisms involved in invasion are still unknown, but some proteins, such as RON2, TRAP, and MSA-1, have been studied for their role in the initial contact and tight binding with erythrocytes [37,38,39]. Each approach has shown its own advantages and disadvantages, which has spurred the search for other vaccine options that confer robust and long-lasting immunity. Therefore, to date, no efficient and effective vaccine against these parasites has been developed [35].

## 5. Conclusions

The ideal concentration of PVP-40 as a cryoprotectant is 20%, which proved to be highly effective in allowing for the efficient revival and recovery of *B. bovis* and *B. bigemina* when cultured in vitro. Vaccinating cattle with cryopreserved *Babesia* parasites in 20% PVP-40 also provided protection against a field challenge where the animals were exposed to ticks infected with *B. bovis* or *B. bigemina*. This research provides valuable insights into vaccine development, but it also has some limitations. For instance, the study involved a small number of animals to test the doses of the parasites that were cryopreserved in a solution containing 20% PVP-40. Another limitation is the short-term immune response during the vaccination and challenge phases. It remains uncertain whether other *Babesia* vaccine strains exhibit different responses to those of wildtype strains sourced from the local farm used in this study. More extensive research is essential to fully addressing and overcoming these limitations. At present, the use of attenuated vaccines derived from in vitro cultures has shown remarkable effectiveness in safeguarding cattle from devastating outbreaks of bovine babesiosis. However, to unlock even greater potential, further research is needed to deepen our understanding of the bovine immune response to these live attenuated vaccines. This vital knowledge will pave the way for the development of more robust and efficient vaccines, ultimately enhancing our ability to prevent bovine babesiosis and protect these invaluable animals. Although advances have been reported using omics, an effective non-live vaccine against babesiosis in cattle has not been developed, but the advancements presented in this study represent a significant step forward in safeguarding cattle health and ensuring the stability of the livestock industry.

## Figures and Tables

**Table 1:** Primer sequence used to amplify *Babesia bovis* and *B. bigemina*.

		Primer Sequence	Amplicon Size
*B. bovis*	Single	CGAGGAAGGAACTACCGATG	354 bp
		GGAGCTTCAACGTACGAGGT	
	nPCR	TGGCTACCATGAACTACAAGACTTA	275 bp
		GAGCAGAACCTTCTTCACCAT	
*B. bigemina*	Single	GAGTCTGCCAAATCCTTAC	879 bp
		TCCTCTACAGCTGCTTCG	
	nPCR	AGCTTGCTTTCACAACTCGCC	412 bp
		TTGGTGCTTTGACCGACGACAT	

nPCR: nested PCR; bp: base pairs.

**Table 2:** Detection of *B. bovis* and *B. bigemina* by nPCR in vaccinated cattle kept on a tick-free farm.

	Days Post-Vaccination				
	8 Days		17 Days		
Treatment Group	*B. bovis*	*B. bigemina*	*B. bovis*	*B. bigemina*	*B. bovis* and *B. bigemina*
Group I	4/6	3/6	5/6	5/6	4/6
Group II	3/6	1/6	3/6	3/6	3/6
Group III	0/5	0/5	0/5	0/5	0/5

**Table 3:** Detection of *B. bovis* and *B. bigemina* by nPCR in vaccinated cattle exposed to a natural challenge in a hyperendemic farm.

	Days Post Challenge				
	8 Days		13 Days		
Treatment Group	*B. bovis*	*B. bigemina*	*B. bovis*	*B. bigemina*	*B. bovis* and *B. bigemina*
Group I	6/6	2/6	5/6	4/6	3/6
Group II	6/6	2/6	6/6	3/6	2/6
Group III	5/5	0/5	5/5	3/5	3/6

**Table 4:** Parameters measured during field challenge.

	Group		
Parameter	I	II	III
Fever day 7 (>40 °C)	0/6	0/6	5/5
Reduction hematocrit (%)	24.1	35.5	40
Parasitemia	3/6	4/6	5/5
Severe disease ^a^	0/6	0/6	5/5
Drug treatment	0/6	0/6	5/5
Protection (%) ^b^	100	100	0

Severe disease was characterized by anemia, lethargy, weakness, depression, weight loss, ruminal atony, and fever for three consecutive days. Drug was only administered in animals who had four consecutive days of parasitemia based on blood smears. a and b: indicate significant differences between Group I and Group II versus Group III (*p* < 0.05).

## Data Availability

Data are contained within the article.
